# Supplementary material for: CHAPAO: Likelihood and hierarchical reference-based representation of biomolecular sequences and applications to compressing multiple sequence alignments
Source: PLoS One. 2022 Apr 18;17(4):e0265360. doi: 10.1371/journal.pone.0265360 (PMC9015123; doi:10.1371/journal.pone.0265360)
Supplement: S1 File — (PDF) [file pone.0265360.s001.pdf]

Supplementary Materials for CHAPAO: likelihood and hierarchical reference-based representation of biomolecular sequences and applications to compressing multiple sequence alignments

Md Ashiqur Rahman[1,†], Abdullah Aman Tutul[1,†], Sifat Muhammad Abdullah [1,†], and
Md Shamsuzzoha Bayzid [1,*]

[1]Department of Computer Science and Engineering
Bangladesh University of Engineering and Technology
Dhaka-1205, Bangladesh
[†]These authors contributed equally to this work
[*]Corresponding author: shams_bayzid@cse.buet.ac.bd

These supplementary materials present additional results (Tables S1 - S16).

1 Supplementary data

Table S1: Summary statistics of the alignments in 16S and 23S ribosomal RNA datasets obtained from the Gutell lab [1, 2].

Dataset	No. of sequences	No. of sites	File size
16S.B.All	27,643	6,857	193.1 MB
16S.T	7,350	11,856	88.7 MB
16S.3	6,323	8,716	56.1 MB
16S.E.all	1,938	7,236	14.3 MB
23S.B.All	3,097	7,330	23.1 MB
23S.T	928	17,618	16.6 MB
16S.T	7350	11,856	88.7 MB
16S.M	901	4,722	4.3 MB
16S.M_aa_ag	1,028	4,907	5.1 MB
seed.23S.B	1,679	6,233	10.7 MB
23S.M	278	10,738	3MB
23S.M.aa_ag	263	10,305	2.8 MB
23S.E	117	9,079	1.1 MB
23S.E.aa_ag	144	8,619	1.3 MB

Table S2: Description of the 10 concatenated alignments from avian phylogenomics project [3, 4].

MSA No	File Name	File Size
1	Exon.heterogenous.c12	34.1M
2	Exon.heterogenous.c123	51.2M
3	concatIntronNooutMSAhigh.fasta	58.2M
4	Exon.homogenous.c12	61M
5	Exon.homogenous.c123	91.5M
6	concatIntronNooutMSAlow.fasta	144.4M
7	aln.phy.c1.phy	212.8M
8	aln.phy.c2.phy	212.8M
9	aln.phy.c3.phy	212.8M
10	intron400.nuc.concat.phy	33.2M

Table S3: Performance of various methods on 10 concatenated alignments from avian phylogenomics project [3, 4]. File sizes are given in bytes.

File Number	zip	bzip2	gzip	MFCompress	LZMA	NAF	CHAPAO
1	8.81M	7.72M	8.53M	1.78M	1.14M	7.29M	1.54M
2	13.01M	11.83M	12.52M	5.12M	2.80M	11.17M	7.62M
3	13.54M	12.46M	13.08M	9.76M	5.22M	11.66M	11.97M
4	16.13M	14.84M	15.61M	3.46M	2.00M	13.32M	2.84M
5	23.79M	21.81M	22.89M	8.43M	4.24M	20.38M	10.74M
6	34.58M	32.04M	33.4M	25.58M	13.12M	29.70M	30.77M
7	57.57M	52.87M	55.59M	16.45M	13.90M	48.85M	13.24M
8	57.42M	52.74M	55.41M	14.28M	11.94M	48.52M	10.38M
9	57.47M	52.96M	55.74M	37.43M	25.73M	49.18M	50.1M
10	8.72M	7.95M	8.43M	5.98M	2.98M	7.53M	7.36M

Table S4: Compression times for various methods on the avian intron MSAs. File sizes are given in bytes and running times are given in seconds.

File size range	zip	bzip2	gzip	MFCompress	LZMA	NAF	CHAPAO
36-979.81K	0.056	0.018	0.120	0.350	0.113	0.044	2.784
979.81K-1.96M	0.243	0.093	0.689	0.739	0.612	0.060	12.540
1.96M-2.94M	0.485	0.180	1.504	0.966	1.211	0.072	22.387
2.94M-3.92M	0.677	0.251	1.957	1.261	1.822	0.065	30.709

Table S5: Compression times for various methods on the avian exon MSAs. File sizes are given in bytes and running times are given in seconds.

File size range	zip	bzip2	gzip	MFCompress	LZMA	NAF	CHAPAO
4.87K-197.37K	0.015	0.014	0.019	0.189	0.028	0.045	0.541
197.37K-389.87K	0.036	0.027	0.080	0.296	0.093	0.047	1.868
389.87K-582.37K	0.070	0.043	0.163	0.400	0.168	0.052	3.409
582.37K-774.86K	0.109	0.061	0.253	0.478	0.257	0.056	4.882

Table S6: Compression times for various methods on the avian UCE MSAs. File sizes are given in bytes and running times are given in seconds.

File size range	zip	bzip2	gzip	MFCompress	LZMA	NAF	CHAPAO
86.96K-109.4K	0.022	0.021	0.035	0.214	0.042	0.049	0.740
109.4K-131.85K	0.025	0.019	0.046	0.237	0.051	0.053	1.024
131.85K-154.29K	0.026	0.018	0.050	0.249	0.055	0.051	1.220
154.29K-176.73K	0.029	0.018	0.052	0.257	0.058	0.053	1.327

Table S7: Compression times for various methods on the 1KP plant dataset. File sizes are given in bytes and running times are given in seconds.

File size range	zip	bzip2	gzip	MFCompress	LZMA	NAF	CHAPAO
1.37K-40.63M	0.044	0.068	0.074	0.342	0.210	0.044	10.036
40.63M-81.26M	1.039	3.586	2.569	6.224	11.261	0.393	539.32
81.26M-121.90M	1.507	5.508	3.399	10.011	17.580	0.501	762.035
121.90M-162.53M	1.768	7.156	4.282	14.674	24.605	0.627	1049.133
162.53M-203.16M	1.827	10.981	5.688	18.030	33.023	0.856	1435.439
243.79M-284.42M	2.655	12.923	7.865	24.391	42.973	1.194	1988.797

Table S8: Compression times for various methods on the avian Concatenated Alignment MSAs. Only files with size less than 300 MB are considered here. File sizes are given in bytes and running times are given in seconds.

File size range	zip	bzip2	gzip	MFCompress	LZMA	NAF	CHAPAO
33M-78M	8.07	3.86	32.16	10.03	31.452	0.586	574.10
78M-123M	17.78	7.11	78.37	18.12	71.326	0.969	1704.02
123M-168M	23.40	11.86	86.41	27.52	110.183	1.948	3168.60
168M-213M	42.63	17.40	169.50	49.92	181.398	2.376	5989.16

Table S9: Compression times for CHAPAO with different windows size and overlap size on the avian concatenated alignments MSA dataset. Only files with size less than 300 MB are considered here. File sizes are given in bytes and running times are given in seconds. Here, W and O denote window size and overlap size, respectively.

File size range	CHAPAO (W=5, O=3)	CHAPAO (W=10, O=8)	CHAPAO (W=20, O=18)	CHAPAO (W=30, O=27)
33M-78M	156.38	298.29	574.10	845.13
78M-123M	333.94	667.28	1704.01	3400.94
123M-168M	799.25	1583.13	3168.59	4096
168M-213M	1628.56	3086.29	5989.15	8600.64

Table S10: Compression times for various methods on 16S dataset. Running times are given in seconds.

File	zip	bzip2	gzip	MFCompress	LZMA	NAF	CHAPAO
16S.3	2.268	2.418	7.279	8.703	11.880	0.479	435.189
16S.B.ALL	7.848	11.239	38.461	26.563	35.777	1.092	2012.838
16S.E.ALL	0.709	0.637	1.787	2.436	2.886	0.112	266.453
16S.T	3.003	3.526	10.680	10.784	18.769	0.413	1438.569
16.S.M	0.166	0.172	0.469	0.851	0.969	0.051	73.918
16S.M.aa_ag	0.141	0.203	0.516	1.263	1.125	0.050	104.556

Table S11: Compression times for CHAPAO with different windows size and overlap size on the 16S dataset. Running times are given in seconds. Here, W and O denote window size and overlap size, respectively.

File Name	CHAPAO (W=5, O=3)	CHAPAO (W=10, O=7)	CHAPAO (W=20, O=15)	CHAPAO (W=30, O=20)
16S.B.ALL	602.540	975.061	1645.463	2186.738
16S.T	191.293	303.992	554.392	753.807
16S.3	121.896	201.527	385.115	531.999
16S.E.ALL	30.410	49.777	94.227	129.522
16S.M	10.432	14.458	27.215	38.006
16S.M.aa_ag	11.831	20.199	30.431	41.863

Table S12: Compression times for various methods on 23S dataset. Running times are given in seconds.

File	zip	bzip2	gzip	MFCompress	LZMA	NAF	CHAPAO
23S.E	0.094	0.062	0.221	0.569	0.310	0.030	9.972
23S.E.aa_ag	0.094	0.062	0.214	0.587	0.306	0.040	11.357
23S.M	0.109	0.109	0.273	0.677	0.654	0.048	22.601
23S.M.aa_ag	0.078	0.109	0.234	0.659	0.626	0.044	20.595
23S.B.ALL	1.509	1.232	2.987	4.017	5.242	0.150	207.271
23S.T	0.562	0.627	1.792	2.453	3.804	0.113	112.459
seed.23S.B	0.724	0.641	1.696	2.001	2.241	0.085	84.614

Table S13: Compression times for CHAPAO with different windows size and overlap size on the 23S dataset. Running times are given in seconds. Here, W and O denote window size and overlap size respectively.

File Name	CHAPAO (W=5, O=3)	CHAPAO (W=10, O=7)	CHAPAO (W=20, O=15)	CHAPAO (W=30, O=20)
23S.B.ALL	48.835	91.838	158.075	228.566
23S.T	30.560	49.386	91.543	125.849
seed.23S.B	22.109	36.393	69.911	97.622
23S.M	6.931	9.043	16.634	22.264
23S.M.aa_ag	6.448	8.314	15.311	20.910
23S.E	3.389	4.012	7.502	9.757
23S.E.aa_ag	4.200	4.748	8.950	12.202

Table S14: Performance of CHAPAO with different windows size and overlap size on the 16S dataset. File sizes are given in bytes. Here, W and O denote window size and overlap size, respectively.

File Name	CHAPAO (W=5, O=3)	CHAPAO (W=10, O=7)	CHAPAO (W=20, O=15)	CHAPAO (W=30, O=20)
16S.B.ALL	4M	3.83M	3.427M	3.176M
16S.T	1.835M	1.797M	1.658M	1.583M
16S.3	1.6M	1.532M	1.404M	1.321M
16S.E.ALL	0.502M	0.482M	0.445M	0.424M
16S.M	0.162M	0.156M	0.149M	0.146M
16S.M.aa_ag	0.159M	0.154M	0.148M	0.146M

Table S15: Compression and decompression times of the best methods including MFCompress, CHAPAO, NAF and LZMA on 16S rRNA dataset. Here, c-time and d-time denote compression time and decompression time (in seconds), respectively. Window size = 5 and overlap = 3 were used for CHAPAO.

Dataset	MFCompress		CHAPAO		NAF		LZMA	
	c-time	d-time	c-time	d-time	c-time	d-time	c-time	d-time
16.S.B.All	23.69	26.16	602.540	9.66	1.09	1.66	35.77	1.285
16S.T	11.12	11.09	191.293	3.29	0.41	0.47	18.76	0.645
16S.3	7.37	7.98	121.896	2.53	0.48	0.55	11.88	0.403
16S.E.All	2.29	2.32	30.410	0.72	0.11	0.08	2.88	0.117
16S.M_aa_ag	0.95	0.95	11.831	0.27	0.05	0.06	1.12	0.037
16S.M	0.81	0.84	10.432	0.42	0.05	0.05	0.96	0.033

Table S16: Performance of CHAPAO with different windows size and overlap size on the 23S dataset. File sizes are given in bytes. Here, W and O denote window size and overlap size, respectively.

File Name	CHAPAO (W=5, O=3)	CHAPAO (W=10, O=7)	CHAPAO (W=20, O=15)	CHAPAO (W=30, O=20)
23S.B.ALL	0.543M	0.488M	0.428M	0.405M
23S.T	0.396M	0.374M	0.354M	0.346M
seed.23S.B	0.243M	0.215M	0.198M	0.189M
23S.M	0.113M	0.110M	0.109M	0.110M
23S.M.aa_ag	0.105M	0.103M	0.102M	0.101M
23S.E	82.798K	80.403K	79.275K	80.905K
23S.E.aa_ag	85.028K	82.595K	83.028K	82.681K

References

1. Cannone JJ, Subramanian S, Schnare MN, Collett JR, D'Souza LM, Du Y, et al. The comparative RNA web (CRW) site: an online database of comparative sequence and structure information for ribosomal, intron, and other RNAs. BMC Bioinformatics. 2002;3(1):2.

2. Gutell RR, Larsen N, Woese CR. Lessons from an evolving rRNA: 16S and 23S rRNA structures from a comparative perspective. Microbiology and Molecular Biology Reviews. 1994;58(1):10–26.

3. Jarvis ED, Mirarab S, Aberer AJ, Li B, Houde P, Li C, et al. Whole-genome analyses resolve early branches in the tree of life of modern birds. Science. 2014;346(6215):1320–1331.

4. Zhang G, Li B, Li C, Gilbert MTP, Jarvis ED, Wang J. Comparative genomic data of the Avian Phylogenomics Project. GigaScience. 2014;3(1):26.
